# Quantifying the effect of crop spatial arrangement on weed suppression using functional-structural plant modelling

**DOI:** 10.1007/s10265-016-0807-2

**Published:** 2016-03-21

**Authors:** Jochem B. Evers, Lammert Bastiaans

**Affiliations:** Centre for Crop Systems Analysis, Wageningen University, Droevendaalsesteeg 1, 6708 PB Wageningen, The Netherlands

**Keywords:** Competition for light, Photosynthesis, Plant–plant interactions, Sink strength, Virtual plant, Weed control

## Abstract

Suppression of weed growth in a crop canopy can be enhanced by improving crop competitiveness. One way to achieve this is by modifying the crop planting pattern. In this study, we addressed the question to what extent a uniform planting pattern increases the ability of a crop to compete with weed plants for light compared to a random and a row planting pattern, and how this ability relates to crop and weed plant density as well as the relative time of emergence of the weed. To this end, we adopted the functional-structural plant modelling approach which allowed us to explicitly include the 3D spatial configuration of the crop-weed canopy and to simulate intra- and interspecific competition between individual plants for light. Based on results of simulated leaf area development, canopy photosynthesis and biomass growth of the crop, we conclude that differences between planting pattern were small, particularly if compared to the effects of relative time of emergence of the weed, weed density and crop density. Nevertheless, analysis of simulated weed biomass demonstrated that a uniform planting of the crop improved the weed-suppression ability of the crop canopy. Differences in weed suppressiveness between planting patterns were largest with weed emergence before crop emergence, when the suppressive effect of the crop was only marginal. With simultaneous emergence a uniform planting pattern was 8 and 15 % more competitive than a row and a random planting pattern, respectively. When weed emergence occurred after crop emergence, differences between crop planting patterns further decreased as crop canopy closure was reached early on regardless of planting pattern. We furthermore conclude that our modelling approach provides promising avenues to further explore crop-weed interactions and aid in the design of crop management strategies that aim at improving crop competitiveness with weeds.

## Introduction

### Improving crop competitiveness

Weeds can be classified as the potentially most serious biotic production constraint to agricultural production, while at the same time actual production losses due to weeds do not differ substantially from those caused by pests and diseases (Oerke 2006). The reason is that, so far, weeds can relatively easily be controlled by a combination of primary tillage (the first soil tillage after the last harvest) and chemical means. This situation is however rapidly changing, as due to the increasing interest in low-frequency tillage systems, weed control is increasingly relying on herbicides. As a result of the smaller spectrum of herbicides following stricter regulations for the admission of these compounds, ideal circumstances are created for a rapid development of herbicide resistance. Care should be taken not to be caught in a negative spiral, where a smaller number of herbicides results in an increased risk of herbicide resistance that in turn results in a further reduction of suitable herbicidal compounds. The only way out of this dead-end track is diversification (Walsh and Powles 2007). Cultural weed control practices, often focussed on prevention rather than on an approach aiming at removal of weeds, are an important means of widening the available range of weed management options (Liebman 2001). Taking the life cycle of an annual weed species as a basis, three main principles can be discerned: reducing the size of the weed seed bank, decreasing the recruitment of weed seeds, and improving crop competitiveness (Bastiaans et al. 2008). Whereas the first two options focus on restricting the number of established weed plants that ultimately compete with the crop, the third option focuses on modifying the crop-weed competitive relations to the benefit of the crop. This study focused on this last option.

There is substantial evidence that significant potential exists for improving weed suppression by optimizing the crop canopy. Time of emergence of the crop relative to that of the weed is an important factor, as an early emergence creates an improved access to available resources. Theory on asymmetric competition between individual plants (Freckleton and Watkinson 2001), particularly for light (Weiner 1986), further predicts that small differences in the initial state of individuals may amplify into considerable size and biomass differences in later stages of development. Selection of the largest seeds for seeding, seed priming and transplanting are agronomic measures to create favourable initial size differences between crop and weed plants. Increased crop competitiveness can also be obtained through breeding. For a long time and a wide range of crop species, experiments have been conducted to identify differences in competitive ability among genotypes (Andrew et al. 2015; Lemerle et al. 1996; Mohler 2001). Attention is given to the characteristics responsible for an increased competitiveness and to the heritability of these characteristics (Zhao et al. 2006).

Improved crop competitiveness can also be obtained by modifications at the population level. Agronomic means that make use of this principle are intercropping, like the addition of a competitive second crop to a weakly competitive main crop (Baumann et al. 2000) and seeding at an increased seeding rate (Zhao et al. 2007). A further option is a more uniform crop spatial arrangement. The effect of planting pattern on weed suppression was theoretically predicted by Fischer and Miles (1973) and experimentally shown in a number of studies (Olsen et al. 2005; Weiner et al. 2001). Both the effects of crop density and plant pattern on weed suppression have been explained in terms of the competitive relationships between crop and weed plants (interspecific competition) as well as among crop plants and among weed plants (intraspecific competition). Increasing crop density strengthens the competitive position of the crop, resulting in suppression of weed growth (Benaragama and Shirtliffe 2013). A more spatially uniform distribution of plants, as opposed to a row-structured canopy, maximizes this effect due to the earlier canopy closure associated with uniform plant spacing (Olsen et al. 2012).

### Modelling crop-weed competition for light

To complement experimental studies, questions on the interaction between crop and weed plants have also been addressed using a range of simulation modelling approaches (Deen et al. 2003). Whereas these models differed in the emphasis and precision level with which certain processes are integrated, their general structure often closely resembles that of INTERCOM, the first well-documented model for crop-weed competition (Kropff and Van Laar 1993). Typically, such models simulate growth of the crop and the weed based on ecophysiological principles of light interception, photosynthesis, biomass production and leaf area expansion. Both crop and weed capture light according to exponential light attenuation (Monsi and Saeki 1953) and the light absorbed is converted through photosynthesis into new biomass and subsequently new leaf area (Goudriaan and Van Laar 1994). Intraspecific competition among crop plants as well as among weed plants in such models is implicitly captured by the exponential decline in light interception per unit of leaf area. Interspecific competition between crop and weed is represented by the simultaneous presence of leaf area of both plant types. Spitters and Aerts (1983) developed a routine for distribution of light over competing species, based on vertical leaf area distribution. In their model the canopy is separated into a large number of layers, and light absorption is calculated for each leaf layer, starting from the top. The distribution of absorbed light over species within each layer is then set proportional to the contribution of leaf area of each species in that layer. This model was further improved by weighing the contribution of leaf area of a species by its extinction coefficient (Kropff et al. 1984).

The crop-weed competition models that contain these routines for light competition have proven instrumental in clarifying the dominant role of relative time of emergence between weed and crop in the competition process. Model analysis of a range of experiments even resulted in the development of an alternative descriptive model of yield reduction due to weeds, whereby relative leaf area replaces weed density as an explanatory factor, to account for differences in relative time of emergence (Kropff and Spitters 1991). The same type of model also showed potential for identifying crop traits responsible for weed suppression, as was demonstrated for rice (Bastiaans et al. 1997). The models however lack the precision level to address the effects of planting pattern on plant competition and weed suppression (Bastiaans et al. 2000). The reason is that, in contrast to the consideration of vertical leaf area distribution, these models assume a spatially uniform distribution of leaf area of crop and weed in the horizontal plane. This issue was addressed by Colbach et al. (2014) by simulating individual plants and their spatial distribution as growing cylinders that compete for resources. Here, we study crop-weed competition for light using a modelling approach called functional structural plant (FSP) modelling (Evers 2016; Vos et al. 2010), that explicitly includes plant architecture and the spatial configuration of the crop-weed canopy, and allows for the simulation of individual plants that grow while competing for resources in three-dimensional (3D) space. The interaction between light and the 3D plant canopy is a particularly strong feature of FSP modelling with a rich history of development (Chelle and Andrieu 1999; Oikawa and Saeki 1977; Takenaka 1994) and is used in many studies that use the approach, e.g. for questions in greenhouse and field crop production (Chen et al. 2014; Evers et al. 2010; Sarlikioti et al. 2011), shade avoidance research (De Wit et al. 2012; Gautier et al. 2000) and interaction between a specific weed (sowthistle) and crop (chickpea) species (Cici et al. 2008). In this study, we specifically addressed the question to what extent the suppressive effect of a more uniform spatial arrangement of the crop is superior over more clustered plant arrangements, like a randomized and row-based design. Furthermore, we investigated whether differences between these arrangements depend on crop and weed plant density as well as on relative time of emergence of the weed. A further objective was to determine the applicability of an FSP modelling approach for studying competition for light between crop and weed.

## Materials and methods

To analyse the interaction between crop and weed density, planting pattern and relative time of emergence, we adopted the FSP modelling approach. The principles and concepts of FSP modelling and their applications in plant and crop research have been explained elsewhere in detail (Evers 2016; Prusinkiewicz and Lindenmayer 1990; Vos et al. 2010). In summary, FSP models simulate growth and development of plants over time in three dimensions, as governed by internal physiological processes under the influence of external driving factors such as light. FSP models are typically developed and calibrated at the level of the plant organ (leaf, internode, fruit) and provide output for testing at the level of the individual plant (height, biomass, leaf area) or plant stand (leaf area index, plot biomass). A key concept in FSP models is the inclusion of plant architectural development—the arrangement of organs taking into account organ orientation, curvature and other geometrical aspects, and the way these change in time.

For this study we developed an FSP model using the software platform GroIMP (Hemmerling et al. 2008). The platform is freely available (www.grogra.de) and the FSP model used in this study is available upon request from the authors. The model simulates, at a daily time step, growth and development of a single-stemmed gramineous phenotype, which was used to represent both the crop and the weed species. We chose to make no distinction between the crop and weed species phenotypes, since in that way any differences observed in the simulation output would not be related to differences in e.g. plant architecture or development rate between the plant types. Additionally, the gramineous architecture chosen has many similarities with actual cereal crops and grassy weed species alike, which increases the relevance of the conclusions drawn from the simulation results to real situations.

The virtual plants were composed of four different organ types: leaf, internode, root system, and spike. Leaf sheaths were not separately considered. The spike and the root system functioned solely as sinks for assimilates, while leaf and internode functioned both as sources (suppliers of assimilates through photosynthesis) for the duration of their lifetime, as well as sinks for the duration of their growth. The functioning of these organs in the context of the plant and the canopy is outlined in the sections below.

### Light absorption, photosynthesis and assimilate production

The simulated scene contained a set of light sources to represent the incoming light. The daily course of the sun was represented by an arc of light sources (Evers et al. 2010). Day of the year and location on the globe further determined the location of these light source with respect to the simulated plants; for all simulations we chose day 90 as the starting day and 52° N as the latitude. The diffuse component of the incoming light was approximated by a dome of weak light sources, arranged in rings at different elevations (Chelle and Andrieu 1999; Evers et al. 2010). The intensity of the direct and diffuse light sources was calculated using a mathematical approximation taken from literature (Goudriaan and Van Laar 1994; Spitters 1986; Spitters et al. 1986). The model used the stochastic path tracer principles (Hemmerling et al. 2008) to determine the fate of the rays of photosynthetically active radiation (PAR) emitted by the light sources. This fate was either absorption by, reflection off, or transmission through a plant organ, determined by the values provided for PAR reflectance (10 % for leaf and internode) and transmittance (5 % for leaf and 0 % for internode). Since during each model time step 2.5 M rays were cast into the simulated scene which were all traced individually and their fates determined, this process resulted in organ-level calculation of distribution of PAR absorption in the canopy. At the organ level, the absorbed PAR was used to calculate photosynthesis rate using a commonly used rectangular hyperbolic photosynthesis-light response curve (Goudriaan and Van Laar 1994). To account for differences in light-saturated photosynthesis rates between organs in high light and organs in low light, maximum photosynthesis rate was linked to the fraction of PAR absorbed by the organ relative to the incoming PAR using the curvilinear relation developed in Niinemets and Anten (2009), with maximum photosynthesis rate in high light fixed at 25 µmol CO_2_ m^−2^ s^−1^ and an initial light use efficiency of 0.06 µmol CO_2_ per µmol PAR. Finally, all CO_2_ assimilated by the organs of a plant each day was converted into growth substrates using CO_2_ molar mass and biomass fraction values, and maintenance costs were deducted (Evers et al. 2010). The result was a daily pool of substrates available for organ growth.

### Potential and actual organ growth

Potential organ growth rate was defined as the sink strength of the organ, i.e. the organ demand for growth substrates. This potential organ growth was implemented using the first derivative of the beta growth function (Yin et al. 2003), a bell-shaped relationship between potential growth rate and organ age. Each organ was assigned parameter values for maximum obtainable biomass (leaf: 200 mg, internode: 300 mg, spike: 3000 mg, roots: 3000 mg) and for growth duration (leaf: 110 °Cd, internode: 180 °Cd, spike: 800 °Cd, roots: 1800 °Cd), determining the exact shape of the sink strength curve for each organ type. To determine whether the potential growth rate of an organ could be reached, i.e. whether the demand for substrates could be satisfied, the relative sink strength concept was used (Heuvelink 1996), in which the sink strength of an organ is expressed as a fraction of total plant sink strength. Each time step and for each organ, a fraction of the available substrates equal to the relative sink strength was assigned to the organ for its growth. If this fraction was less than the demand, the organ received the assigned amount but its growth would be lower than the potential growth. If this fraction exceeded organ demand, the organ would reach potential growth and the remaining substrate was stored. The excess growth substrates stored from all organs were made available for growth in the next time step. Finally, the substrates received by each organ were converted into new organ area or length, using a fixed LMA (leaf mass per unit of leaf area) parameter of 3.5 mg cm^−2^ for the leaves, and an SIL (specific internode length) parameter of 0.5 mm mg^−1^ for the internodes. To account for shade-induced additional internode extension, the SIL value was increased for low plant source-sink ratios, following an exponential relationship resulting in unaffected SIL for source-sink ratios above 1, and SIL reaching 10 for extremely limiting conditions (source/sink ratios approaching 0). The net result of this was that variation in height in a canopy of simulated plants was low, as reduced internode length for plants in less favourable conditions was compensated for by a higher SIL, mimicking real shade-induced stem extension. The newly formed leaf area and internode length determined canopy architecture and therefore organ light absorption in the next time step, closing the circle. To initiate growth of the plant, a seed endosperm mass of 30 mg was provided for each plant, enough to form the first leaf area.

The result of this process was that each time step, the organs of a plant were competing for assimilates. Since each time step existing organs aged and new organs were initiated (see *Development and architecture* below), the supply–demand relationships within a plant changed continuously. Ultimately this resulted in a plant consisting of organs of different biomasses and sizes, reflecting the situation the organs experienced during their growth period. Plants competing with one another therefore heavily determined each other’s internal source-sink relationships and as a result the simulated plants displayed a phenotype shaped by their neighbours.

### Development and architecture

In contrast to the mechanistic simulation of plant growth described above, plant development (the creation of new organs) was simulated in a descriptive manner using so-called L-system rewriting rules that form the basis of many FSP models (Prusinkiewicz and Lindenmayer 1990). Plant development was temperature-driven, assuming a constant daily thermal time increment of 15 °Cd.

Each simulated plant was equipped with an apical meristem which was responsible for the production of new organs. Every 45 °Cd (the plastochron) the apical meristem produced a new internode and a new leaf at the top of the stem. Upon creation, organ age increased each time step and the organs started to attract growth substrates. To mimic cereal architecture, the first four internodes had a sink strength of zero and therefore did not grow in length, resulting in the first four leaves to visually emerge from soil level. To represent the increasing delay between leaf creation and leaf appearance with rank commonly observed in gramineous species (McMaster 2005), leaf growth was set to start attracting growth substrates after a delay that increased with leaf rank, resulting in an interval between consecutive leaf appearances (the phyllochron) of 90 °Cd.

After having produced 10 leaves, the apical meristem switched to the generative phase, and produced a peduncle carrying the spike, a strong sink for assimilates. Due to the difference in plastochron and phyllochron, the spike was created while the upper leaves were still acting as sinks, resulting in competition between the spike and the upper leaves for assimilates. Leaves were shed after an age equal to four times their duration of expansion, or when light level at the leaf reached a critical lower threshold of 10 µmol PAR quanta m^−2^ s^−1^, whichever of the two occurred first. In the three-dimensional scene, the leaves were represented by narrow oblong surfaces with a shape typical for gramineous leaves (eq. A1 in Evers et al. 2006) with the maximum width located at 62 % of the leaf length from the leaf tip, a curvature coefficient of 0.76 and a length–width ratio of 25. Leaves were homogeneously curved at an angle of 100° between the tangents at the leaf base and the tip. Leaf orientation was determined by a phyllotactic angle of 137° (rotation angle between two consecutive leaves) and an insertion angle of 40° (angle between leaf base and stem). The internodes were represented by cylinders at a maximum width of 5 mm.

### Field setup

**Fig. 1:**
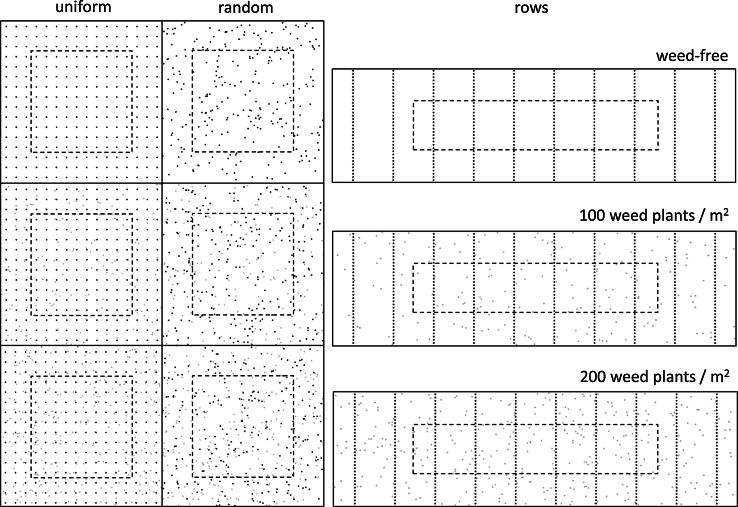
Graphical representation of the field setups used in this study for a crop plant density of 200 plants m^−2^. The plant patterns are uniform (*left column*), random (*middle column*), and rows (*right column*). Weed infestation levels range from weed-free (*top row*), 100 weed plants m^−2^ (*middle row*) to 200 weed plants m^−2^ (*bottom row*). Each *dot* indicates the position of a plant. Crop plant positions are represented by the *black dots*, and weed plant positions are represented by the *grey dots*. Plant positions outside of the *dashed area* were treated as *border* plants. Model output was based only on the focal plants within the *dashed area*. The *black solid* borders represent the size of the simulated fields. For the crop density of 400 plants m^−2^, the crop plants were more closely spaced and therefore fields were proportionally smaller. The positions of the weed plants as well as the positions of the crop plants in the random plant pattern were chosen randomly each simulation run

Three different crop plant arrangements were tested in this study: uniform, random and rows (Fig. 1). In the uniform arrangement, the plants were placed in a 16 by 16 square grid at equal distance between the plants in four directions. To account for border effects, the outer three plants on all four sides were not taken into account, leaving 100 focal plants to be used for output calculation. Full plant emergence was set to occur at a random day within a time window of 3 days. Two crop plant densities were tested (200 and 400 plants m^−2^) by adapting distance between the plants. This resulted in total plot sizes of 1.28 and 0.64 m^2^ including the border plants, respectively, and net plot sizes of 0.5 and 0.25 m^2^ for the focal plants only. For the random plant arrangement, identical field and border sizes were used as in the uniform pattern, but plant positions were randomized within the limits of the entire plot. Finally, in the row pattern, plants were arranged in 10 rows of 35 plants each. Two rows on either side of the plot were regarded as border rows, and 10 plants on either side of the rows were regarded as border plants, resulting in 90 focal plants. The two densities were reached by setting row distance to 25.0 cm (200 plants m^−2^) and 17.7 cm (400 plants m^−2^), and keeping the ratio between plant and row distance constant at 0.08. This resulted in total plot sizes of 1.75 and 0.87 m^2^ including the border plants, respectively, and net plot sizes of 0.45 and 0.22 m^2^ for the focal plants only. Weeds were introduced by randomly placing weed plants in the simulated plot at a density of either 100 or 200 weed plants m^−2^, and emergence of the weeds was set to occur either in the same time window as the crop plants, 3 days earlier, or 3 days later. Note that to maintain weed densities, the absolute number of simulated weed plants at a certain weed density was lower at the high than at the low crop density, due to the differences in plot size between the two crop densities.

### Simulations

To test the effects of relative time of weed emergence, weed density and crop density on the competitive relations between crop and weed plants at different planting patterns, a complete factorial simulation design was adopted in which all variable levels were tested against each other. In total, two crop densities (200 and 400 plants m^−2^), three weed densities (0, 100 and 200 plants m^−2^), three planting patterns (random, row, uniform) and three relative times of weed emergence (minus 3 days, simultaneous, plus 3 days) were tested. Since relative time of emergence could obviously not be tested for the weed-free situation, this resulted in 42 combinations in total. Due to the stochastic elements in the model each simulation was run three times, adding up to 126 simulations in total. Model stochasticity was caused by the stochasticity inherent to the light model, the random placement of plants where applicable, the randomly chosen orientation of individual plants, and the randomly chosen time of emergence within the range set. Simulations were run for 90 time steps, representing 90 days. Note that in the situation of a random crop plant arrangement at 200 plants m^−2^ with weeds emerging simultaneously at a density of 200 plants m^−2^, there was no difference between crop and weed.

### Output and analysis

Output was generated at the level of the plot, taking into account the focal plants only. For the crop and the weed, leaf area index, daily assimilated CO_2_ and aboveground biomass was saved each time step. Weed biomass at 90 DAS was used to determine the influence of planting pattern on the competitive ability of the crop. This analysis was conducted for the three emergence dates of the weed separately. Simulated data were fitted to a rectangular hyperbola describing the relation between weed biomass (Y_w_; g m^−2^) and densities of weed (N_w_; plants m^−2^) and crop (N_c_; plants m^−2^) according to Spitters (1983):

1 $$Y_{w} = \frac{{N_{w} }}{{b_{w0} + b_{ww} N_{w} + b_{{wc_{ran} }} N_{{c_{ran} }} + b_{{wc_{row} }} N_{{c_{row} }} + b_{{wc_{uni} }} N_{{c_{uni} }} }}$$

In this equation, the effect of interspecific competition of a crop in each of the planting patterns (*ran* is random, *row* is row planting, *uni* is uniform) is represented by the product of an interspecific competition coefficient (b_wc_; m^2^ g^−1^) and crop plant density. Note that for any of the simulated cases plant density of at least two patterns was set to zero, as all together only one planting pattern was present at the same time. Parameter b_w0_ (plant g^−1^) represents the reciprocal of single plant biomass if weed plant density approaches 0 and b_ww_ (m^2^ g^−1^) represents the intraspecific competition coefficient for weed plants. Data on weed biomass of a specific emergence date were simultaneously fitted to weed and crop plant density, using the non-linear regression option of GENSTAT 17^th^ edition (VSN International, Hempstead, UK). This analysis provided values for the interspecific competition coefficients for all three planting patterns, facilitating a direct comparison of the competitive ability of the crop between spatial configurations.

## Results

### Crop LAI, assimilation, and final biomass

**Fig. 2:**
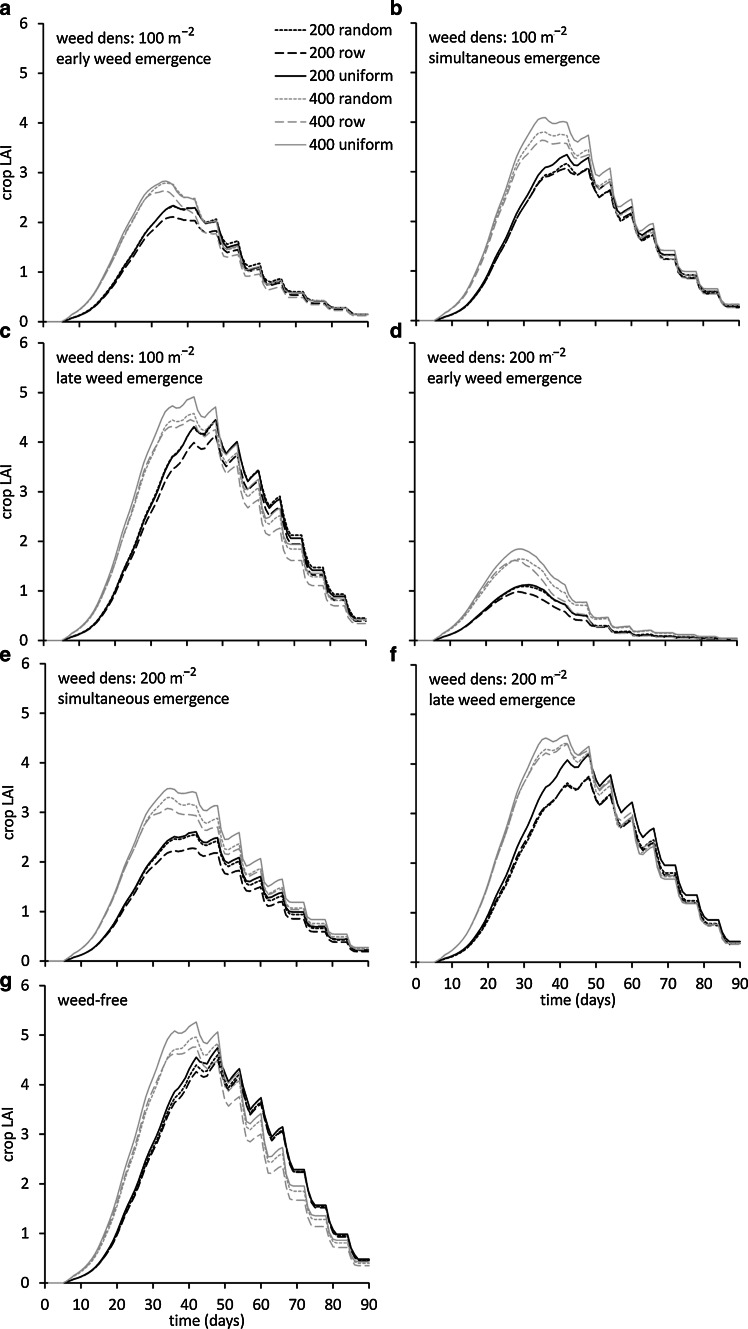
Simulated crop LAI over time as affected by weed plant density of 100 (**a**, **b**, **c**) and 200 (**d**, **e**, **f**) plants m^−2^ as well as by weed emergence relative to the crop, either early (3 days; **a**, **d**) simultaneous (**b**, **e**) or late (3 days; **d**, **f**) for crop densities of 200 (*black lines*) and 400 (*grey lines*) plants m^−2^ and three planting arrangements (random: *dotted lines,* row: *dashed lines*, uniform: *solid lines*). In (*g*), crop LAI in the absence of weeds is presented

**Fig. 3:**
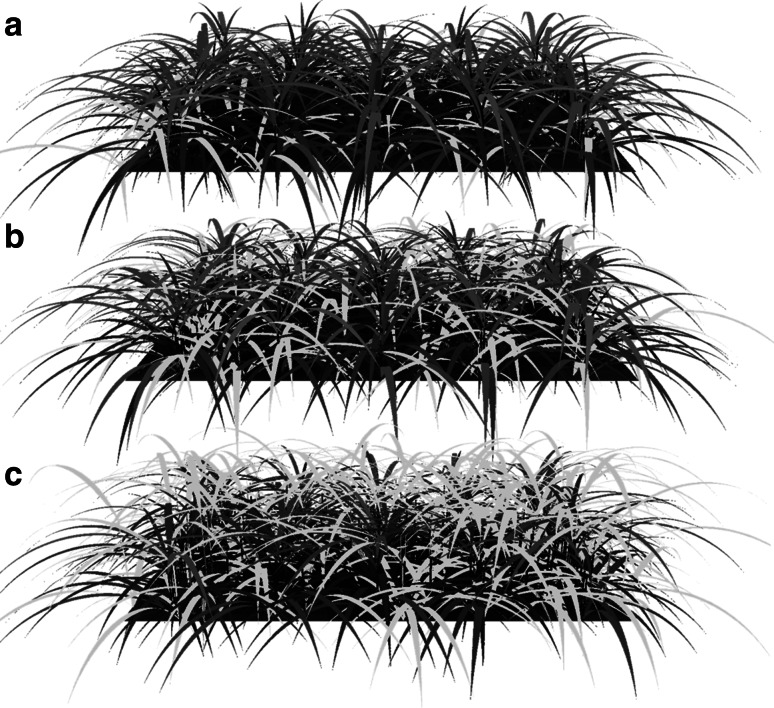
Example of the visual output of the model showing the 37-day stage of a setup with crop plants (*dark leaves*) in the *row* arrangement at 200 plants m^−2^ and weed plants (*bright leaves*) at 100 plants m^−2^ for late weed emergence (**a**), simultaneous weed emergence (**b**) and early weed emergence (**c**). Note that for clarity of the figure, only 5 crop *rows* are shown instead of the 10 used in the simulations

The build-up of crop leaf area over time was predominantly affected by the relative time of weed emergence (Fig. 2): early weed emergence resulted in maximum crop LAI of between 2.0 for 200 crop plants m^−2^ and 3.0 for 400 crop plants m^−2^ at a weed density of 100 plants m^−2^. The difference between emergence dates is visually demonstrated in Fig. 3 for 37 days after emergence and shows the suppressive effect of the crop on leaf area of the weed plants (Fig. 3a) and vice versa (Fig. 3c) in the case of the row arrangement. Maximum LAI was even further reduced at a weed density of 200 plants m^−2^ to values between 1.0 for 200 crop plants m^−2^ and 2.0 for 400 crop plants m^−2^. Similarly, a delayed weed emergence resulted in higher maximum crop LAI values between 4 and 5 at 100 weed plants m^−2^ and between 3.5 and 4.5 at 200 weed plants m^−2^. In contrast, planting pattern showed marginal effects on LAI, with the row pattern giving slightly lower maximum LAI values in a number of cases.

**Fig. 4:**
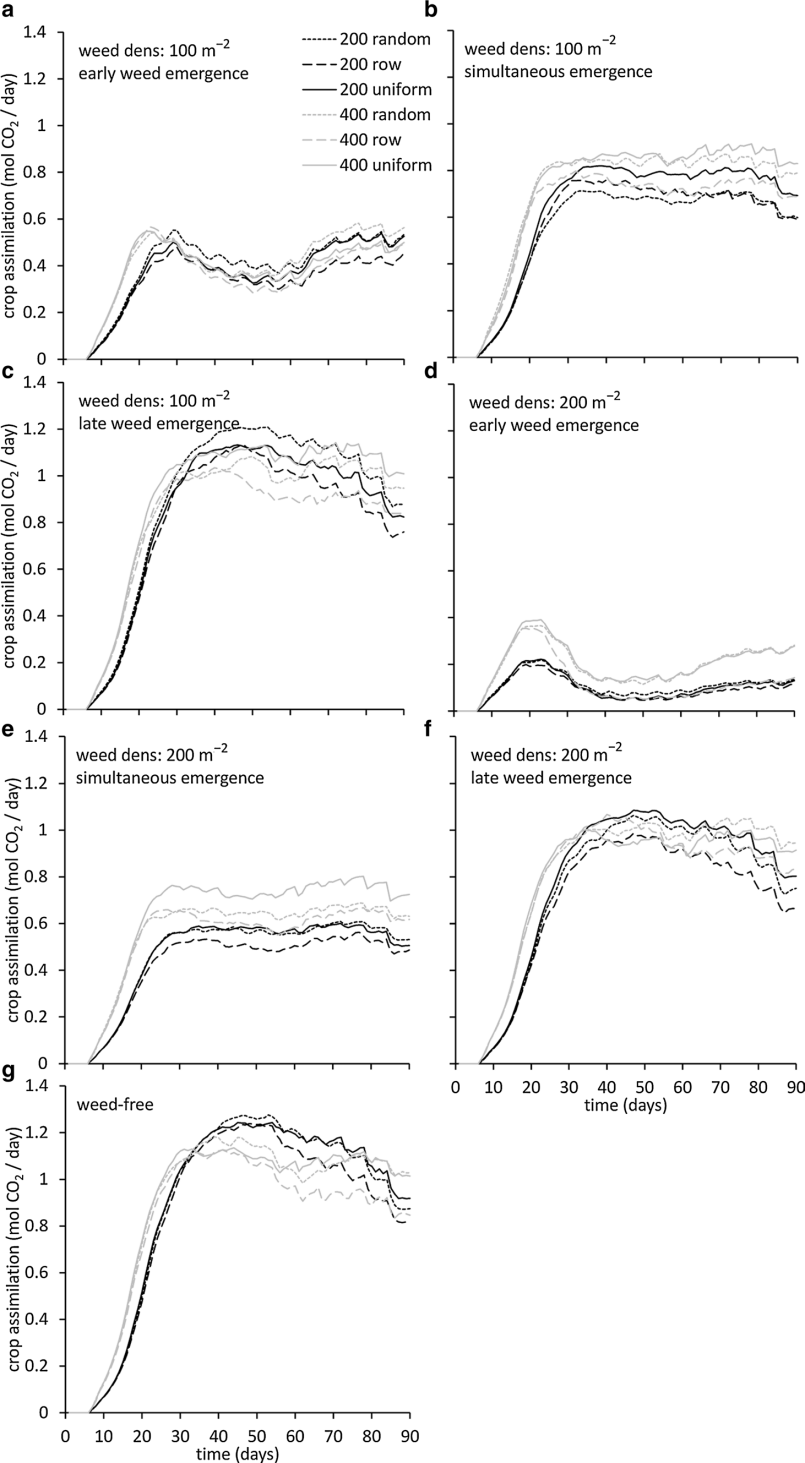
Simulated crop daily assimilation (mol CO_2_ day^−1^) over time as affected by weed plant density of 100 (**a**, **b**, **c**) and 200 (**d**, **e**, **f**) plants m^−2^ as well as by weed emergence relative to the crop, either early (3 days; a, d) simultaneous (b, e) or late (3 days; d, f) for crop densities of 200 (*black lines*) and 400 (*grey lines*) plants m^−2^ and three planting arrangements (random: *dotted lines*, row: *dashed lines*, uniform: *solid lines*). In (*g*), crop daily assimilation in the absence of weeds is presented

A key driver of the suppressive effect of weed emergence time on crop leaf development was the reduction in photosynthesis and associated CO_2_ assimilation rate by the weed plants (Fig. 4). A head start for the weed plants resulted in considerable suppression of crop assimilation rate beyond approximately 25 days (Fig. 4a, d), which impeded further exponential leaf area growth and resulted in the lower maximum LAI values observed. At simultaneous emergence the suppressive effect of the weed plants on crop assimilation can be observed in the plateau in assimilation rate occurring beyond approximately 30 days (Fig. 4b, e). Only at late weed emergence the crop plants reached high assimilation rates (Fig. 4c, f). Consistent differences between planting patterns could not be observed in crop assimilation rates, but differences did appear to be more prominent compared to those in LAI. Also here the row setup showed moderately lower assimilation in a number of the scenarios, but results were not consistent.

In the end, final aboveground biomass produced confirmed the observations on LAI and assimilation during crop and weed growth (Table 1). Final biomass was severely affected by relative emergence date and by weed density, but only moderately to not at all by planting pattern, depending on the exact combination of settings. In many cases, the row pattern resulted in the lowest final crop biomass. This was also observed for the weed-free situation, which indicates a direct effect of planting pattern on intraspecific competition between crop plants in the absence of weeds.

**Table 1:** Aboveground biomass at 90 DAS of the cereal and the weed plants at the three planting arrangements for all combinations of weed relative emergence, weed plant density and crop plant density (*n* = 3)

Plant type	Weed relative emergence	Weed density (plants m^−2^)	Crop density (plants m^−2^)	Random arrangement		Row arrangement		Uniform arrangement	
				Biomass (g m^−2^)	S.E.	biomass (g m^−2^)	S.E.	Biomass (g m^−2^)	S.E.
Cereal	Early	100	200	380.4	26.6	336.6	13.9	375.3	20.8
			400	392.4	32.5	337.0	32.1	378.1	17.9
		200	200	84.0	12.3	73.6	8.7	81.9	9.2
			400	135.2	35.1	90.5	10.5	146.5	42.6
	Simultaneous	100	200	600.5	2.9	586.7	30.7	637.1	34.3
			400	683.5	51.7	640.3	17.7	736.9	29.6
		200	200	471.2	24.4	425.2	22.5	484.4	29.0
			400	568.5	21.4	543.1	14.1	624.0	35.8
	Late	100	200	924.4	28.7	825.2	23.3	895.7	7.1
			400	890.5	19.1	797.1	18.8	935.9	31.7
		200	200	765.5	36.2	740.6	7.5	850.1	12.4
			400	848.4	33.1	817.5	14.2	848.3	11.4
	Weed-free	0	200	962.1	21.0	925.9	6.8	989.0	13.4
			400	912.5	32.2	819.5	39.2	960.8	13.1
weed	Early	100	200	606.9	7.8	625.6	34.2	595.9	66.0
			400	544.9	29.6	578.8	44.5	508.0	18.0
		200	200	861.1	28.0	834.1	29.1	885.3	27.8
			400	850.5	31.9	785.4	45.7	761.4	94.3
	Simultaneous	100	200	295.4	17.4	315.5	24.5	277.0	36.5
			400	192.0	33.0	170.7	7.7	148.8	18.7
		200	200	425.0	25.3	412.9	20.2	395.4	31.9
			400	280.9	22.4	228.0	27.7	243.9	4.5
	Late	100	200	48.2	11.8	45.6	3.4	32.2	3.2
			400	5.5	1.0	5.0	0.3	6.8	0.4
		200	200	110.3	24.7	103.8	9.1	74.7	13.4
			400	15.6	4.7	14.0	4.4	11.7	1.1

### Crop competitiveness

Weed biomass obtained at 90 DAS was used to estimate interspecific competition coefficients for the three planting patterns using Eq. 1. For each relative emergence time an adequate description of weed biomass in dependence of weed and crop plant density was obtained, with percentage variance accounted for exceeding 95 %. The increase in competition parameter values with later emergence of the weed displays the more profound suppressive effect of crop plants on weeds that emerged relatively late (Table 2). However, focal point of the current study was the effect of crop spatial arrangement on weed suppression by the crop. For each relative emergence time the ranking of the interspecific competition parameters was the same, with weed-suppression ability of the crop being highest in the uniform arrangement, followed by the row setup, and finally the random arrangement. With early emergence of the weed, the suppressive effect of crop plants was smallest, whereas the differences between planting patterns were largest. The ratio between the competition parameter of a uniform and a random plant arrangement at early weed emergence was for instance 1.459. This implies that crop plant density would need to be 45.9 % higher in the random pattern for the crop to have an equal suppressive effect on the weeds as in the uniform placement at early weed emergence. Interestingly, the differences between the three planting patterns became smaller if the weed emerged later. With equal emergence, the difference between uniform and random planting was still 15.2 %, whereas with late weed emergence the difference further dropped to 7.1 %. For these three emergence times of the weed, the interspecific competition coefficient of crop plants in a uniform pattern was 27.4, 8.4 and 6.1 % higher than that of the row pattern. Consequently, an initial 14.6 % advantage of the row configuration over the random planting with early weed emergence, diminished to 6.3 % at equal emergence and almost completely disappeared (0.9 %) with late emergence of the weed.

**Table 2:** Estimates of competition parameters and their standard errors for three different crop spatial arrangements at three different relative times of weed emergence (3 days before the crop, simultaneous, and 3 days after the crop)

	Early weed emergence		Simultaneous emergence		Late weed emergence	
	*b*_*wc*_	S.E.	*b*_*wc*_	S.E.	*b*_*wc*_	S.E.
random	0.0774	0.0269	1.32	0.136	62.4	3.6
row	0.0887	0.0273	1.41	0.138	62.9	3.6
uniform	0.113	0.0282	1.52	0.142	66.8	3.6
	*b*_*wc*_/*b*_*wc*_	S.E.	*b*_*wc*_/*b*_*wc*_	S.E.	*b*_*wc*_/*b*_*wc*_	S.E.
row/random	1.146	0.273	1.063	0.0636	1.009	0.0024
uniform/row	1.274	0.273	1.084	0.0673	1.061	0.0052
uniform/random	1.459	0.348	1.152	0.0714	1.071	0.0056

Parameter estimates (*b*_wc_ in m^2^ g^−1^) were obtained by fitting Eq. 1 to simulated weed biomass at 90 DAS, expressed in absolute values, 10^3^ and in ratios

## Discussion

### Uniform planting improves weed suppression particularly in the absence of a head start for the crop

Our results indicated that planting pattern did not have a large overall effect on the crop: uniform spacing between plants, randomized plant position, or a highly aggregated structure in rows all appeared to result in a similar leaf area development of the crop and comparable crop assimilation rates. In contrast, relative time of emergence of the weed as well as weed density had a considerable impact on crop performance, corroborating earlier experimental and modelling work (Cousens et al. 1987; Kropff et al. 1992). The effect of crop plant density was particularly evident during the first half of the growing period and was reflected in a faster increase in leaf area and crop assimilation rate at higher density (Figs. 2, 4). In the second half of the growing period these differences disappeared. Despite its minimum effect on crop performance, planting pattern did affect the suppressive effect of crop plants on weeds (Table 2). Regardless of relative emergence time of the weed, a uniform planting pattern always resulted in more competitive crop plants, whereas a random planting pattern resulted in the least competitive crop canopy. Relative emergence time did however influence the actual competitive strength of crop plants and also had a strong impact on the differences in weed suppressiveness among planting patterns. Interestingly, the random pattern resulted in the least competitive crop, even though the row pattern showed the lowest crop aboveground biomass (Table 1) in all simulations. This emphasizes that the capacity of the crop to suppress weeds is not necessarily fully related to crop production, due to the interaction with other canopy characteristics such as planting pattern. This shows the relevance of the FSP modelling approach for studying crop-weed competition, which is resulting from its ability to accommodate differences in leaf positioning in 3D space.

Our analysis provided new insights in how relative time of emergence interacted with the differences in weed suppressiveness among planting patterns. Differences where strongest with an early emergence of the weed and considerably smaller when the crop was given a head start relative to the weed. Extrapolating these results to an even wider time frame than the 6-days-difference in time of emergence used in this simulation study suggests that with a large enough head start of the crop, differences in weed suppression among planting pattern will disappear altogether. In this situation the crop is able to form a closed canopy, regardless of planting pattern, long before the weeds can exert a negative effect on the crop. With a narrowing of the time gap between crop and weed emergence, the uniform planting pattern is better able to express its ability to produce a closed canopy in the shortest possible period of time. This is because for a long period of time neighbouring plants hardly overlap, implying that all newly produced leaf area contributes maximally to canopy closure. Secondly, because postponement of this overlap minimizes intraspecific competition among crop plants, the exponential growth phase of the crop canopy is extended. In contrast, in the situation when the crop emerges after weed establishment, the crop plants are outcompeted. Consequently, crop plants might remain so small that intraspecific competition among crop plants, at least in a uniform planting pattern, is further postponed or never occurs at all. It thus creates conditions where the differences between uniform and more clustered patterns are as large as possible. However, from an agronomic perspective this situation is not relevant at all, due to the strong negative effect of the weed on crop production, following from the head start of the weed.

The strong reliance on herbicidal control is threatening the sustainability of current weed management systems. Development of sustainable integrated weed management systems requires alternative measures to allow for diversification. In this regard, cultural weed control measures, defined as small adjustments in the general management of the crop that contribute to the regulation of weed populations and reduce the negative impact of weeds on crop production, are important. Adequate control based on these measures will, however, only be obtained when applied in combination, a phenomenon generally referred to as the strategy of the ‘many little hammers’ (Liebman and Gallandt 1997). Improving crop competitiveness is one of the principles behind a range of cultural weed control measures, that also include a uniform planting pattern. The current study reinforced that a uniform spatial arrangement indeed increases the weed-suppression ability of crop plants. However, the present study also revealed that the superiority of a uniform planting pattern decreases if crop plants are given a head start relative to the weeds. The implication is that in combination with measures that create a favourable starting position for the crop, like seed priming or transplanting, the advantage of a uniform planting pattern is sub-optimal. Such information is highly relevant for the design of alternative weed management strategies.

### Functional-structural plant modelling as a platform for crop-weed interaction analysis

The approach we developed has potential to analyse crop-weed interactions at a precision level that has not been possible with previous (modelling) approaches. Although some explicitly include the spatial distribution of individual plants in crop-weed canopies (Colbach et al. 2014), our method explicitly takes into account competition for light between individual plants with a realistic representation of plant architecture, and scales up photosynthesis and growth at the organ level to the level of the plant stand. Intra- and interspecific competition between plants for light is an emergent property of the model. Due to the spatially explicit nature of the approach, the effects of plant arrangement on competitive interactions between plants can be taken into account, since the immediate environment of a plant is shaped by the positions and architecture of its neighbours (Iwaki 1959). Our results suggest that considering individual plants is particularly relevant during the early stages of crop development, before the crop has developed a closed canopy. For crop-weed competition these early stages are crucially important, as it is during these phases of crop development that the competitive relations between crop and weed are shaped. After the exponential growth phase the individual crop plants gradually become subordinated within the population and resource supply becomes the main determinant of dry matter production.

Clearly, our approach is only a first step towards a deeper analysis of crop-weed interactions at the plant level. There are a number of limitations that warrant elaboration in future studies. The current approach only considered the aboveground parts of the plant. In principle, the approach is very well suited to include the root system and soil environment and its resources (Dunbabin et al. 2013; Pagès and Picon-Cochard 2014). This would allow for a more comprehensive study of competition for resources both above and belowground (light, nutrients, water), and potentially for the inclusion of weed seed production (Bastiaans et al. 2000). Furthermore, the approach allows for the analysis of the effect of different architectural or physiological phenotypes on the interaction between crop and weed. For instance, a weed species with a broadleaf architecture and a less vertical growth pattern is likely to have different competitive interactions with a cereal-type crops species than the grassy weed architecture used in the current study. Similarly, species with distinctly different physiological characteristics could be tested in competition. Finally, a relevant extension to the current approach would be to include plastic shade avoidance responses to neighbouring plants (Bongers et al. 2014; De Wit et al. 2012; Evers et al. 2007; Zhu et al. 2015). These responses allow a plant to anticipate future competition and grow foliage in those places where neighbour shading is expected to be minimal. Maize for instance redirects leaf growth to areas away from neighbouring plants (Maddonni et al. 2002). It might well be that, due to these responses, differences in weed-suppression ability among planting patterns are actually smaller if crop and weed plants have the plasticity to respond to the availability of open patches caused by planting pattern. This reinforces that theoretical studies like the one we presented here require experimental verification. Nevertheless, the approach is very promising, as it allows exploring processes in plant–plant interaction, helps to identify potentially interesting options to be tested experimentally and permits to quantify and compare the relevance of various cultural weed control options.
